# Continuous control of endotracheal cuff pressure and tracheal wall damage: a randomized controlled animal study

**DOI:** 10.1186/cc6142

**Published:** 2007-10-03

**Authors:** Saad Nseir, Alexandre Duguet, Marie-Christine Copin, Julien De Jonckheere, Mao Zhang, Thomas Similowski, Charles-Hugo Marquette

**Affiliations:** 1 Intensive Care Unit, Calmette Hospital, University Hospital of Lille, boulevard du Pr Leclercq, 59037 Lille cedex, France; 2 Intensive Care Unit, Department of Respiratory Diseases, Public Hospitals of Paris, La Pitié-Salpêtrière Hospital, 47-83 boulevard de l'Hôpital, 75013 Paris, France; 3 Department of Pathology, Biology and Pathology Center, University Hospital of Lille, Lille 2 University, 1 place de Verdun, 59045 Lille, France; 4 Institut de Technologie Médicale, EA1049, CHRU de Lille, Pavillon Vancostenobel, 2 avenue Oscar Lambret, 59037 Lille cedex, France; 5 Department of Emergency Medicine, Zhejiang University, School of Medicine and Research Institute of Emergency Medicine, Zhejiang University, Hangzhou, China; 6 Respiratory Disease Department, Calmette Hospital, University Hospital of Lille, boulevard du Pr Leclercq, 59037 Lille cedex, France

## Abstract

**Background:**

Intubation is frequently performed in intensive care unit patients. Overinflation of the endotracheal tube cuff is a risk factor for tracheal ischemia and subsequent complications. Despite manual control of the cuff pressure, overinflation of the endotracheal cuff is common in intensive care unit patients. We hypothesized that efficient continuous control of the endotracheal cuff pressure using a pneumatic device would reduce tracheal ischemic lesions in piglets ventilated for 48 hours through a high-volume, low-pressure endotracheal tube.

**Materials and methods:**

Twelve piglets were intubated and mechanically ventilated for 48 hours. Animals were randomized to manual control of the endotracheal cuff pressure (*n *= 6) or to continuous control of the endotracheal cuff pressure using a pneumatic device (*n *= 6). In the two groups, we inflated the endotracheal cuff with 50 ml air for 30 minutes, eight times daily. This hyperinflation of the endotracheal cuff aimed at mimicking high-pressure periods observed in intubated critically ill patients. In all animals, the cuff pressure and the airway pressure were continuously recorded for 48 hours. After sacrifice of the study animals, the trachea was removed and opened longitudinally for gross and histological examination. A pathologist evaluated the slides without knowledge of treatment group assignment.

**Results:**

The cuff pressure was significantly lower in piglets with the pneumatic device than in piglets without the pneumatic device (median (interquartile range), 18.6 (11–19.4) cmH_2_O versus 26 (20–56) cmH_2_O, *P *= 0.009). No significant difference was found in the percentage of time spent with a cuff pressure <15 cmH_2_O and that with a cuff pressure between 30 and 50 cmH_2_O. The percentage of time between 15 and 30 cmH_2_O of cuff pressure, however, was significantly higher in piglets with the pneumatic device than in piglets without the pneumatic device (98% (95–99%) versus 65% (44–80%), *P *= 0.002). In addition, the percentage of time with cuff pressure >50 cmH_2_O was significantly lower in piglets with the pneumatic device than in piglets without the pneumatic device (0% versus 19% (12–41%), *P *= 0.002).

In all animals, hyperemia and hemorrhages were observed at the cuff contact area. Histological examination showed no difference in tracheal lesions between animals with and without the pneumatic device. These lesions included deep mucous ulceration, squamous metaplasia and intense mucosal inflammation. No cartilage lesions were observed.

**Conclusion:**

The pneumatic device provided effective continuous control of high-volume, low-pressure endotracheal cuff pressure in piglets mechanically ventilated for 48 hours. In the present model, however, no significant difference was found in tracheal mucosal lesions of animals with or without a pneumatic device. Further studies are needed to determine the impact of continuous control of cuff pressure over a longer duration of mechanical ventilation.

## Introduction

Endotracheal intubation is frequently performed in intensive care unit (ICU) patients [1]. The endotracheal tube cuff is responsible for tracheal mucosal lesions that are visible at the cuff contact area a few hours after intubation [2-5]. These lesions may result in serious complications such as tracheal stenosis and tracheal ruptures [6-8]. According to the results of studies using a low-volume, high-pressure endotracheal cuff, the prevalence of postintubation and post-tracheotomy stenosis varies from 10% to 19% in ICU patients [9,10]. More recent studies using a high-volume, low-pressure cuff, however, showed that clinically significant stenosis was less common (1‰–1%) [11,12]. Hyperinflation of the endotracheal tube cuff is the most frequent risk factor for tracheal ischemia and subsequent complications in these patients [13].

Complications related to insufficient cuff inflation have nevertheless been reported, including leaking of the tidal volume and microaspiration of secretions and subsequent ventilator-associated pneumonia [14]. In most ICUs, the endotracheal cuff pressure is never checked [15-18]. In these ICUs, caregivers frequently overinflate the tube cuff to prevent gas leak and pulmonary aspiration [15,18].

High-volume, low-pressure endotracheal tubes have significantly reduced the frequency of ischemic tracheal lesions. Even when high-volume, low-pressure endotracheal tubes are used, however, ischemic tracheal lesions may occur [19]. An endoscopic study performed in 40 patients undergoing surgery showed that obstruction of mucosal blood flow occurred at a lateral wall pressure above 30 cmH_2_O [20].

Based on recent recommendations, the cuff pressure should be maintained around 25 cmH_2_O in critically ill intubated and mechanically ventilated patients [21,14]. Although manual measurement of the cuff pressure could reduce overinflation and underinflation frequency, manual measurement may not provide effective control of the cuff pressure. As shown by Duguet and colleagues [22], despite manual control of the endotracheal pressure with a portable manometer according to the French Society of Critical Care Medicine recommendations, the percentage of time the cuff pressure was >30 cmH_2_O was 29 ± 25% and the percentage of time the cuff pressure was <15 cmH_2_O was 15 ± 17%.

Several devices enabling efficient continuous control of cuff pressure have been recently described [22,23]. The pneumatic device is a simple mechanical device that continuously maintains the cuff pressure during mechanical ventilation with minimal human resources [22].

We hypothesized that efficient continuous control of the endotracheal cuff pressure using a pneumatic device would reduce tracheal ischemic lesions in piglets ventilated for 48 hours through a high-volume, low-pressure endotracheal tube.

## Methods

This study was conducted in the experimental intensive care unit at Lille II University. All animals were treated according to the guidelines of the Department of Experimental Research of Lille University and according to the *Guide for the Care and Use of Laboratory Animals *(NIH Publication Number 93-23, revised 1985).

### Animal preparation

Healthy, bred, domestic Largewhite-Landrace piglets, weighing 22 ± 2 kg, were anesthetized using propofol 3 mg/kg and were orotracheally intubated with a 7.0 Hi-Lo Lanz™ Malinckrodt tube (Malinckrodt Inc, Argyle, NY, USA). Anesthesia was maintained with a continuous infusion of midazolam 0.3 mg/kg/hour, pancuronium 0.3 mg/kg/hour and fentanyl 0.3 μg/kg/hour. The femoral artery was cannulated with a 3 F polyethylene catheter (Plastimed, St Leu la Forêt, France) for pressure monitoring. An 8 F suprapubic urinary catheter (Vesicoset; Angiomed, Karlsruhe, Germany) was placed in the bladder transabdominally. Animals were mechanically ventilated in the prone position in a volume-controlled mode with a Cesar type 1 ventilator (Taema, Antony, France). The ventilatory parameters consisted of a tidal volume of 15 ml/kg, a respiratory rate of 15 breaths/minute, an expiratory ratio of 0.5 and zero end-expiratory pressure. Inspired gases were humidified using a conventional humidifier (MR290; Fisher Paykel, Auckland, New Zealand), and an initial fraction of inspired oxygen of 0.21 was used. All animals were sacrificed 48 hours after starting mechanical ventilation.

### Device for control of endotracheal cuff pressure

**Figure 1:**
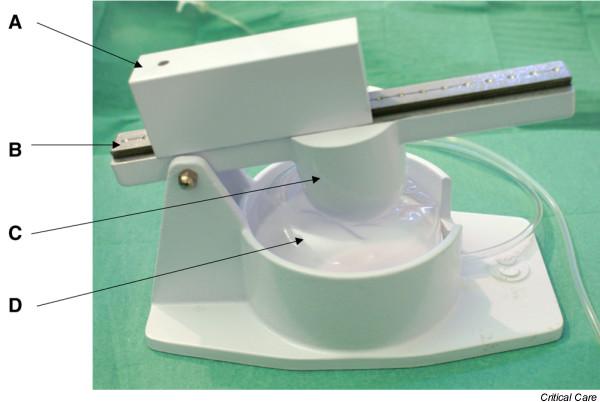
Photograph of the pneumatic device. A, mobile mass; B, arm; C, fixed mass; D, 200 ml cuff connected to the external control cuff of the endotracheal tube.

The Nosten^® ^device (Leved, St-Maur, France) is amechanical appliance that does not require a power supply (Figure 1). Asterile single-use 200 ml cylindrical cuff encased in arigid compartment is connected to the endotracheal cuff with plastic tubing (internal diameter 3 mm, length 2 m). Aweight mounted on an articulated arm constantly exerts pressure on this cuff. This pressure can be adjusted by moving another weight along the arm to modulate the corresponding force, allowing the user to obtain the desired cuff pressure. Any variation is immediately cancelled out by the disproportion between the volumes of the two cuffs [22]. The device provides effective continuous control of endotracheal cuff pressure in mechanically ventilated ICU patients [22].

### Study protocol

Twelve animals were randomly assigned (1:1) to one of the two study groups. In the interventional study group, the endotracheal cuff was connected to the continuous cuff pressure control device, the mobile weight of which was moved along the articulated arm to obtain acuff pressure of 22 cmH_2_O. In the standard care group, the cuff pressure was managed according to the French Society of Intensive Care recommendations [24]; namely, a target cuff pressure at 22 cmH_2_O with cuff pressure checks twice a day at fixed intervals and after each intervention on the endotracheal tube (manual portable manometer, Hi-Lo™; Tyco Healthcare, Hazelwood, Mo, USA).

**Figure 2:**
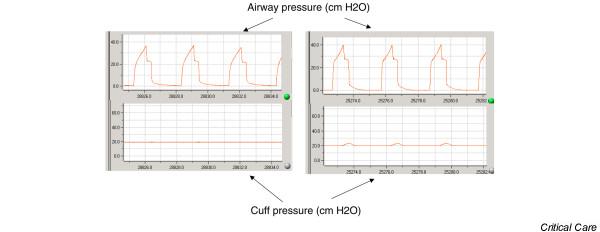
Continuous recording of cuff and airway pressures in piglets with and without the pneumatic device. Left: continuous recording of the cuff pressure and the airway pressure in a piglet with the pneumatic device – the cuff pressure was constant despite variations of airway pressure. Right: continuous recording of the cuff pressure and the airway pressure in a piglet without the pneumatic device – the cuff pressure decreased and increased with airway pressure variations.

In all animals, the cuff pressure and the airway pressure were continuously recorded at adigitizing frequency of 100 Hz for 48 hours (Physiotrace^®^; Estaris, Lille, France) (Figure 2) [25]. The connection between the pressure transducer and the endotracheal cuff was identical in the two groups, with athree-way stopcock of which the third port was either closed or connected to the pneumatic device. During each experiment, two piglets were randomized to the standard care group or to the pneumatic device group. Continuous recording of the cuff pressure and the respiratory pressure was performed simultaneously in the two animals. Connections were checked every 3 hours.

In the two groups, we inflated the endotracheal cuff with 50 ml air for 30 minutes eight times daily. This hyperinflation of the endotracheal cuff aimed at mimicking high-pressure periods observed in intubated critically ill patients [22]. After each period of hyperinflation, the cuff pressure was readjusted as described above. Hyperinflation periods represented 16% of the total duration of mechanical ventilation (8 hours out of the total 48 hours).

### Postmortem evaluation

**Figure 3:**
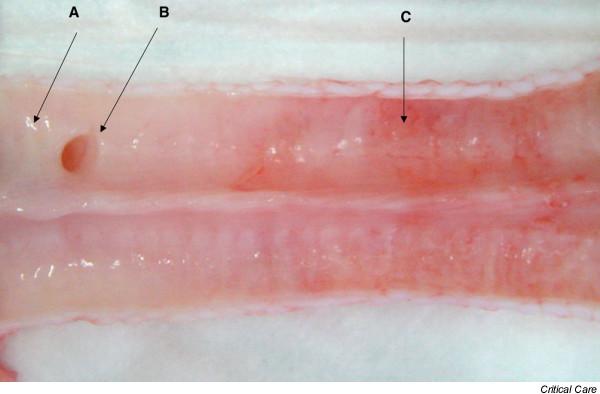
Gross examination of longitudinally opened trachea. A, no lesions on tracheal mucosa distal to the endotracheal tube; B, origin of the tracheal bronchi; C, hyperemia and hemorrhages at the cuff contact area.

After sacrifice of the study animals, the trachea was removed and opened longitudinally for gross examination. Full-thickness samples of two contiguous tracheal rings were collected and were placed in formalin for later histological examination. The first sample was taken from the mid-cuff contact area, and the second sample was taken distally beyond the endotracheal tube. The proximal limit of cuff contact with mucosa was easily recognized in all animals by visual examination of the tracheal mucosa (Figure 3). The pathologist evaluated the slides without knowledge of treatment group assignment. Tracheal lesions were graded as: Grade I lesions including squamous metaplasia, few inflammatory cells, and edema; as Grade II lesions including mucous ulceration and normal subcartilaginous tissue; or Grade III lesions including mucous ulceration and a dense inflammatory reaction from the surface tissue to the subcartilaginous tissue [7].

### Statistical analysis

SPSS software (SPSS, Chicago, IL, USA) was used for data analysis. In each animal, we measured the time spent with a cuff pressure below 15 cmH_2_O, a pressure between 15 and 30 cmH_2_O, a pressure between 30 and 50 cmH_2_O, and with a cuff pressure over 50 cmH_2_O. Qualitative variables were described as the number (percentage), and quantitative variables were described as the median (interquartile range). The distribution of quantitative values was tested for normality using the Shapiro–Wilk test. Proportions were compared using the chi-square test or the Fisher exact test where appropriate. The Student *t *test or the Mann–Whitney U test was used for quantitative variables, as appropriate. Differences were considered significant if *P *< 0.05. We expected grade II or grade III tracheal lesions would occur in all control animals. Inclusion of 12 animals (six in each group) was required to detect a difference of 60% in the rate of animals with grade II or grade III tracheal lesions (two-sided α = 0.05, power = 0.80).

## Results

The mean arterial pressure (100 (85–110) mmHg versus 100 (89–115) mmHg), the diastolic arterial pressure (70 (61–80) mmHg versus 68 (59–78) mmHg) and the heart rate (101 (90–115) beats/min versus 98 (89–112) beats/min) were similar (*P *> 0.2) in animals with the pneumatic device and in animals without the pneumatic device.

The mean airway pressure was similar in piglets with or without the pneumatic device (11.3 (11–12.5) cmH_2_O versus 12.4 (10.4–13.2) cmH_2_O, *P *= 0.5). The cuff pressure was significantly lower in piglets with the pneumatic device than in piglets without the pneumatic device (18.6 (11–19.4) cmH_2_O versus 26 (20–56) cmH_2_O, *P *= 0.009). During overinflation periods, the cuff pressure was significantly lower in piglets with the pneumatic device than in piglets without the pneumatic device (23 (20–25) cmH_2_O versus 76 (63–82) cmH_2_O, *P *< 0.001). No significant difference was found in the percentage of time spent with a cuff pressure <15 cmH_2_O and the percentage of time with a cuff pressure between 30 and 50 cmH_2_O. The percentage of time between 15 and 30 cmH_2_O cuff pressure, however, was significantly higher in piglets with the pneumatic device than in piglets without the pneumatic device. In addition, the percentage of time >50 cm H_2_O cuff pressure was significantly lower in piglets with the pneumatic device than in piglets without the pneumatic device (Table 1).

**Table 1:** Endotracheal cuff pressure in animals with and without the pneumatic device

	Animals with the pneumatic device (*n *= 6)	Animals without the pneumatic device (*n *= 6)	*P *value
Percentage of time at <15 cmH_2_O	1.4 (0.02–4.3)	0.3 (0.02–22.9)	0.910
Percentage of time at 15–30 cmH_2_O	98 (95–99)	65.8 (44–80)	0.002
Percentage of time at 30–50 cmH_2_O	0.01 (0–0.02)	0.3 (0–0.95)	0.315
Percentage of time at >50 cmH_2_O	0	19.8 (12–41)	0.002

Results presented as the median (interquartile range).

Macroscopic examination showed no lesions on the tracheal mucosa distal to the endotracheal tube. In all animals, however, hyperemia and hemorrhages were observed at the cuff contact area (Figure 3).

**Figure 4:**
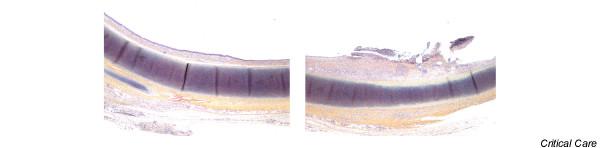
Histological examination (1 × 2.5) of tracheal samples. Left: sample taken distally beyond the endotracheal tube, no visible lesions. Right: sample taken from the cuff contact area with localized ulceration.

**Figure 5:**
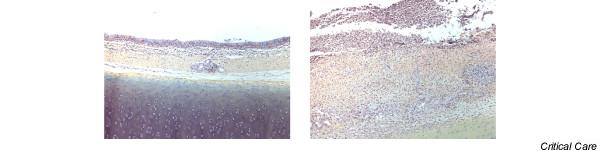
Histological examination (1 × 10) of tracheal samples. Left: sample taken distally beyond the endotracheal tube showing moderate inflammation. Right: sample taken from the cuff contact area with localized ulceration, including fibrin and polynuclear cells, squamous metaplasia and intense mucosal inflammation.

Histological examination showed no difference in tracheal lesions between animals with or without the pneumatic device. Although no lesions were observed in samples taken distally beyond the endotracheal tube, grade I and grade II lesions were observed in all animals in samples taken from the cuff contact area (Table 2). These lesions included deep mucous ulceration, including fibrin and polynuclear cells, squamous metaplasia and intense mucosal inflammation. Neither cartilage lesion nor inflammation expanding to the subcartilaginous tissue was observed (Figures 4 and Figure 5).

**Table 2:** Distribution of histological tracheal lesions

	Animals with the pneumatic device (*n *= 6)	Animals without the pneumatic device (*n *= 6)
Grade I lesions	6 (100)	6 (100)
Grade II lesions	6 (100)	6 (100)
Grade III lesions	0 (0)	0 (0)

Results presented as *n *(%).

## Discussion

In piglets ventilated for 48 hours through a high-volume, low-pressure endotracheal tube, the pneumatic device enabled an effective continuous control of the endotracheal cuff pressure. This effective control of cuff pressure did not, however, result in any difference with regard to tracheal mucosal damage.

Continuous recording of the cuff pressure in study animals confirmed that the pneumatic device was efficient at continuous cuff pressure regulation. The high volume of the pneumatic-device cuff (200 ml) explains how the injection of 50 ml air did not result in endotracheal cuff overinflation in animals with the pneumatic device, since the endotracheal cuff and the pneumatic-device cuff were connected during inflation periods. In a previous prospective study, the efficacy of the pneumatic device in maintaining constant endotracheal cuff pressure was evaluated in nine consecutive mechanically ventilated critically ill patients [22]. The cuff pressure was continuously registered for 24 hours during standard care and for 24 hours with the regulatory device. The authors reported a significant reduction in the coefficient of variation of cuff pressure in patients during the period of mechanical ventilation with the pneumatic device. Other devices are available for cuff pressure control [23,26-28]; however, the device used in the present study has the advantage of being extremely simple to use. In addition, it contains no electronics and does not depend on any sort of power supply.

Despite effective control of the cuff pressure with the pneumatic device, no difference was found in tracheal ischemia between animals with the pneumatic device and those without. This observation suggests that continuous control of the cuff pressure is not effective in preventing tracheal wall damage for a short duration (≤ 48 hours) of mechanical ventilation through a high-volume, low-pressure endotracheal tube. The severity of tracheal damage, however, is related to the duration of intubation [20]. Further studies should therefore determine whether continuous control of the endotracheal cuff pressure could reduce the severity of tracheal ischemia over a longer duration of mechanical ventilation. One potential explanation for the absence of a relationship between effective control of the endotracheal cuff pressure and tracheal mucosal lesions is the fact that the cuff pressure in piglets without the pneumatic device was relatively low. If a higher cuff pressure had been used in control animals, a histological difference might have been observed. Our study design aimed at mimicking the clinical situation in intubated and mechanically ventilated ICU patients with manual control of the cuff pressure. In most ICU patients, however, the cuff pressure is never checked [15,16]. This suggests that the cuff pressure was probably lower in control group than in patients without manual control of cuff pressure. Another possible explanation for the absence of significant difference in histological lesions was the short duration (30 min, eight times daily) of hyperinflation periods in our study. In a clinical setting, hyperinflation periods may occur for longer duration, especially when the cuff pressure is never checked.

In a prospective experimental study, Touzot-Jourde and colleagues [29] randomly assigned orotracheally intubated anesthetized horses to an endotracheal cuff pressure of 80–100 cmH_2_O or 120 cmH_2_O. Although the duration of invasive mechanical ventilation was short (175 ± 15 min), the tracheal damage was found to be more severe and occurred more frequently in the higher cuff pressure group. The cuff pressures used in their study, however, were much higher than those used in our study. In a study performed in patients with short duration of intubation and mechanical ventilation [30], higher cuff pressure was also associated with a significantly higher rate of ischemic tracheal lesions diagnosed by fiberoptic examination.

Large-volume, low-pressure endotracheal tube cuffs are claimed to have a less deleterious effect on tracheal mucosa than high-pressure, low-volume cuffs. Low-pressure cuffs could easily be overinflated, however, to yield pressures that will exceed capillary perfusion pressure resulting in impaired mucosal blood flow. Loeser and colleagues [19] found a much reduced mean depth of erosion in dogs intubated with large-volume, low-pressure cuffed tubes inflated to the clinical seal for periods of 5–7 hours; however, the area of erosion was significantly greater with the large volume cuff. Impairment of tracheal mucosal blood flow is an important factor in tracheal morbidity associated with intubation. Hence it is recommended that a cuff inflation pressure of 30 cmH_2_O (22 mmHg) should not be exceeded to prevent tracheal wall damage [20]. In a study performed in intubated rabbits, superficial tracheal damage occurred within 15 minutes at lateral wall pressure of 27 cmH_2_O. There was partial denuding of the basement membrane with a lateral wall pressure of 68 cmH_2_O. At a lateral wall pressure of 136 cmH_2_O, damage extended to the basement membrane and mucosal stroma within 15 minutes – and this damage was progressive with time [31]. The prone position was used in our study since in pigs, as in sheep or cows, mechanical ventilation in the supine position results in lung atelectasis with severe ventilation/perfusion mismatch after a few hours [32]. Whether these results are applicable in animals ventilated in the supine position is unknown. In addition, our results were obtained in healthy piglets. Tracheal lesions could therefore have been more important if animals had prior tracheal inflammation.

Some limitations of our study should be taken into account. First, animals were intubated and mechanically ventilated for only 48 hours. Our results therefore may not be applicable for a longer duration of mechanical ventilation. Second, the small number of animals that were studied is another limitation of the present study. Larger studies with longer exposure of the tracheal mucosa to cuff overinflation could therefore demonstrate a beneficial effect of the pneumatic device in reducing ischemic tracheal lesions. Third, inflation of the endotracheal cuff with 50 ml air may have been excessive as compared with clinical practice. This maneuver, however, aimed to generate high endotracheal cuff pressures, which are difficult to obtain with small volumes of air when high-volume, low-pressure tubes are used. By contrast, using smaller volumes of air is associated with similar cuff pressures when low-volume, high-pressure tubes are used. The high cuff pressures recorded during inflation periods (>70 cmH_2_O) in control animals were similar to those used in previous animal studies to evaluate tracheal mucosal lesions [20,31]. Another reason for the use of such a high volume of air was to test the efficacy of pneumatic device in preventing cuff overinflation.

## Conclusion

We conclude that the pneumatic device provides an effective continuous control of the endotracheal cuff pressure in intubated and mechanically ventilated piglets. No difference was found, however, in tracheal mucosal lesions between animals with or without the pneumatic device. Our results suggest that continuous control of the endotracheal cuff pressure within the recommended pressure range does not necessarily prevent tracheal ischemia, at least in piglets ventilated for 48 hours with a high-volume, low-pressure endotracheal tube. Further studies are needed to determine the impact of continuous control of the cuff pressure over a longer duration of mechanical ventilation.

## Key messages

• The pneumatic device provides effective continuous control of endotracheal cuff pressure in intubated and mechanically ventilated piglets.

• No difference was found in tracheal mucosal lesions between animals with or without the pneumatic device.

• Our results suggest that continuous control of endotracheal cuff pressure within the recommended pressure range does not necessarily prevent tracheal ischemia, at least not in piglets ventilated for 48 hours with a high-volume, low-pressure endotracheal tube.

• Further studies are needed to determine the impact of continuous control of the cuff pressure over a longer duration of mechanical ventilation.

## Abbreviations

ICU = intensive care unit.

## Competing interests

The authors declare that they have no competing interests.

## Authors' contributions

SN, AD, TS, and C-HM designed the study. SN and MZ performed the animal experiments. M-CC performed the histological examination. JDJ performed analysis of the cuff and airway pressure recording. SN wrote the manuscript, and all authors participated in its critical revision. SN had full access to all data in the study and had final responsibility for the decision to submit for publication. All authors read and approved the final manuscript.
